# Changing the Cortical Conductor’s Tempo: Neuromodulation of the Claustrum

**DOI:** 10.3389/fncir.2021.658228

**Published:** 2021-05-13

**Authors:** Kelly L. L. Wong, Aditya Nair, George J. Augustine

**Affiliations:** ^1^Neuroscience and Mental Health Program, Lee Kong Chian School of Medicine, Nanyang Technological University, Singapore, Singapore; ^2^Institute of Molecular and Cell Biology (IMCB), Agency for Science, Technology and Research (A*STAR), Singapore, Singapore; ^3^Computation and Neural Systems, California Institute of Technology, Pasadena, CA, United States

**Keywords:** claustrum, acetylcholine, serotonin, dopamine, neuromodulation

## Abstract

The claustrum is a thin sheet of neurons that is densely connected to many cortical regions and has been implicated in numerous high-order brain functions. Such brain functions arise from brain states that are influenced by neuromodulatory pathways from the cholinergic basal forebrain, dopaminergic substantia nigra and ventral tegmental area, and serotonergic raphe. Recent revelations that the claustrum receives dense input from these structures have inspired investigation of state-dependent control of the claustrum. Here, we review neuromodulation in the claustrum—from anatomical connectivity to behavioral manipulations—to inform future analyses of claustral function.

## Introduction

The claustrum is a long and irregular sheet of neurons nestled between the insula and striatum. As it is known to be heavily and bilaterally connected to many brain regions in organisms ranging from mice to humans (Sherk, 1986; Torgerson et al., 2015; Wang et al., 2017, 2019; Zingg et al., 2018), the claustrum has been likened to a cortical conductor (Crick and Koch, 2005). Despite being anatomically described since the late 1700s, even now little is known about claustral function. This is largely due to difficulties in reliably targeting the claustrum for experimental analysis. Recently, application of molecular targeting approaches that permit reliable experimental interrogation of the claustrum has greatly advanced our understanding of the claustrum (Jackson et al., 2020). Current evidence points to the claustrum being involved in higher cognition. Hypothesized functions of the claustrum revolve around four main themes: consciousness (Crick and Koch, 2005; Koubeissi et al., 2014; Chau et al., 2015; Yin et al., 2016; Bickel and Parvizi, 2019), attention and salience (Mathur, 2014; Chia et al., 2017; Atlan et al., 2018; Smith et al., 2019b), learning and memory (Grasby and Talk, 2013; Jankowski and O’Mara, 2015; Renouard et al., 2015; Liu et al., 2019; O’Mara and Aggleton, 2019; Reus-García et al., 2020), and sleep (Renouard et al., 2015; Narikiyo et al., 2020; Norimoto et al., 2020).

Brain states are coordinated changes in brain-wide activity observed during conditions such as wakefulness, sleep and anesthesia. They are thought to result from the actions of neuromodulators, such as acetylcholine (ACh), dopamine (DA) and serotonin (5-HT), that affect brain processes ranging from macroscopic networks down to subcellular signaling (Kringelbach and Deco, 2020; McCormick et al., 2020). The claustrum, like every other brain region, may be regulated by different combinations of neuromodulators during different brain states. Thus, it is difficult to determine the precise function of the claustrum without considering how different brain states influence the claustrum.

Given that the claustrum is interconnected with many brain regions, it is poised to participate in large-scale networks that orchestrate wakefulness, sleep, anesthesia and other cognitive functions. Yet it is entirely unclear how the claustrum operates in an ever-changing neuromodulator landscape: How does the claustrum conductor change its “tempo”, namely the way it controls the cortex? In this review article, we build upon on the previous review of Baizer (2014) by providing an updated and more comprehensive view of what is known about modulation of the claustrum by ACh, DA and 5-HT. We also briefly consider other neuromodulators and define important factors for thinking about neuromodulation of the claustrum. Finally, we provide a unified view of neuromodulator control of the claustrum, along with a simplified model for the unique role of the claustrum in brain function.

## Ach and The Claustrum

Involvement of the cholinergic system in attention is well known: activation of cholinergic neurons in the basal forebrain recapitulates many of the effects of attention on cortical firing rate and rate variability (Minces et al., 2017; Schmitz and Duncan, 2018). Therefore, interplay between the basal forebrain cholinergic system and the claustrum has also been hypothesized to enable the claustrum’s role in attention (Goll et al., 2015). However, whether the cholinergic system implements its role in attention in concert with the claustrum has not been experimentally tested. Here, we review evidence for cholinergic modulation of the claustrum and highlight one recent study from our group that addresses potential mechanisms of cholinergic modulation through a cell-type specific effect in the claustrum.

### Evidence of Cholinergic Input and Receptors

**Figure 1:**
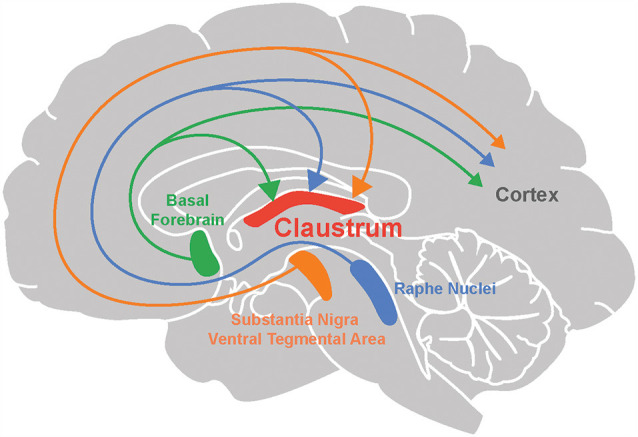
Neuromodulation of the claustrum. The claustrum, as well as the cortex, receives neuromodulatory input from the basal forebrain (acetylcholine), raphe nuclei (serotonin), substantia nigra and ventral tegmental area (dopamine).

The cholinergic system emanating from the basal forebrain sends diffuse projections throughout the brain (Ballinger et al., 2016). Many recent studies provide anatomical evidence for cholinergic input to the claustrum (Figure 1; Atlan et al., 2018; Zingg et al., 2018; Narikiyo et al., 2020). Monosynaptic retrograde tracing, using rabies viruses to target claustral neurons that project to the retrosplenial cortex, reveal that cholinergic input is likely to be the largest source of neuromodulatory input to the claustrum (Zingg et al., 2018). This study also observed that although both cholinergic neurons and GABAergic neurons in the basal forebrain project to the claustrum, the former constitute a larger percentage of claustrum-projecting neurons. Studies based on claustrum-specific, Cre-based mouse lines also find an abundance of cholinergic input to the claustrum. Using anterograde and retrograde viral tracing in the Egr2-claustrum line, Atlan et al. (2018) identified the substantia inominata (SI) of the basal forebrain as the major cholinergic nucleus projecting to Egr2-expressing neurons in the claustrum. This finding was corroborated in another mouse line, Tbx21-Cre, by Narikiyo et al. (2020), who also identified the SI as a major source of cholinergic input to the claustrum.

The basal forebrain cholinergic system exerts its influence throughout the brain by activating both of the main types of ACh receptors (AChRs; Ballinger et al., 2016). One type consists of muscarinic ACh receptors (mAChRs), which are metabotropic and thus activate G proteins to alter the gating and kinetics of K^+^, Ca^2+^ and non-selective cation channels. The second type consists of nicotinic ACh receptors (nAChRs), which are ionotropic and are non-selective cation channels that are opened by binding of ACh. AChRs are diverse, with at least 5 mAChR subtypes and an even larger number of nAChR subunits that combinatorially assemble into pentameric receptors that vary in their location and precise signaling roles (Luchicchi et al., 2014; Ballinger et al., 2016).

Not all types of AChR have been found in the claustrum. Studies have noted a particularly high expression of α4, α5 and α7 nAChR subunits and M4 mAChRs in the claustrum of mice (Gong et al., 2003; Han et al., 2003; Winzer-Serhan and Leslie, 2005). There is also evidence for high expression of choline acetyltransferase (ChAT), an enzyme required for ACh synthesis, in the primate claustrum (Sutoo et al., 1994). Thus, the molecular machinery for ACh signaling is present in the claustrum.

### Cholinergic Modulation of the Claustrum Microcircuit

Despite the wealth of anatomical and histological evidence for cholinergic modulation, little is known about how ACh affects claustrum neuron function. The first study was done by Salerno et al. (1981), who investigated the actions of ACh within the claustrum by iontophoretically applying ACh onto neurons of the cat claustrum. These experiments showed that ACh exerted heterogeneous effects, exciting 40% and inhibiting 47% of the 92 claustrum neurons examined. However, this pioneering work did not distinguish between direct and polysynaptic effects of ACh application.

The role of endogenous cholinergic signaling has recently been clarified in a study by our group that used optogenetics to probe cholinergic modulation in mouse claustrum brain slices (Nair et al., 2021). Optogenetic activation of cholinergic axons indicated that claustral neurons receive cholinergic input in a cell-type specific manner: cortex-projecting claustrum neurons were primarily inhibited, while claustrum neurons projecting to subcortical structures (including thalamus and habenula) and vasointestinal peptide (VIP) expressing interneurons in the claustrum were excited. The excitation of VIP interneurons in the claustrum mirrors observations in the cortex (Fu et al., 2014), with the notable exception that cholinergic input did not excite somatostatin (SST) expressing interneurons or parvalbumin (PV) expressing interneurons in the claustrum (Nair et al., 2021) but does excite these interneurons in the cortex.

The excitatory responses to cholinergic input observed by Nair et al. (2021) were monosynaptic and mediated by nAChRs, in line with the high expression of nAChRs in the claustrum noted above (Winzer-Serhan and Leslie, 2005). Nair et al. (2021) observed that inhibitory cholinergic input received by cortex-projecting claustral neurons was also monosynaptic but was produced by a surprising co-release of ACh and GABA by the basal forebrain. Thus, depending on their projection targets, claustrum neurons receive opposing excitatory and inhibitory input from the basal forebrain.

Cholinergic modulation is known to alter the gain of cortical neurons, which is defined as a change in neuronal input-output properties (Silver, 2010; Polack et al., 2013; Dasilva et al., 2019). Similarly, cholinergic input also affects the gain of claustral neurons (Nair et al., 2021). The opposing excitatory and inhibitory effects observed on different projection neuron subtypes in the claustrum translates into opposing effects on the gain of these neurons: cholinergic input decreases the gain of cortex-projecting claustral neurons, while increasing the gain for subcortex-projecting neurons and VIP interneurons. This serves to enhance the ability of cortex-projecting neurons to distinguish strong from weak excitatory postsynaptic potentials, as well as strong postsynaptic potentials from noise. A claustrum network model predicts that cholinergic input acts as a switch to toggle information processing between the cortex-projecting and subcortex-projecting neurons of the claustrum. In a non-cholinergic state, information flow in the claustrum is more efficient for the subcortex-projecting neurons, while cholinergic input switches information flow towards the cortex-projecting neurons of the claustrum.

### Cholinergic Modulation as a Permissive Gate for the Claustrum in Attention and Sleep

Recent findings have converged on a critical role for the claustrum in attention (Atlan et al., 2018; Fodoulian et al., 2020). These studies demonstrate that inactivation of the claustrum impairs an animal’s performance in attention-related tasks and makes them more susceptible to distractors in their environment. This may be mediated by indirect inhibition of the cortex, *via* claustrum neurons that excite cortical interneurons (Atlan et al., 2018; Jackson et al., 2018), although recent work by Fodoulian et al. (2020) suggests that it could also be mediated, in part, by direct excitation of the cortex. The inhibition observed by Atlan et al. (2018) reduced cortical gain and was suggested to suppress the representation of irrelevant stimuli in the environment.

How might cholinergic modulation contribute to the claustrum’s role in attention? We propose that cholinergic modulation is a permissive agent: in attentive states, when cholinergic input is high, the observations of Nair et al. (2021) predicts that information flow will be biased towards the cortex-projecting claustrum population, allowing this projection to inhibit the cortex. In non-attentive states, when cholinergic levels are low, the claustrum will be unable to inhibit cortical targets as information flow is toggled towards the subcortex-projecting claustrum neurons (Nair et al., 2021). This proposal is consistent with research that has focused on the role of claustrocortical connections in attention (Atlan et al., 2018; Jackson et al., 2018; Fodoulian et al., 2020). In addition, regions such as the thalamic reticular nucleus and central thalamus have been reported to contribute to aspects of attention, including sensory selection and attentional effort (Schiff et al., 2013; Wimmer et al., 2015). More research must be done to determine whether claustrothalamic projections and their modulation by cholinergic input also contribute to attention.

Such a permissive role for cholinergic modulation may also explain the claustrum’s role in sleep. Studies by Narikiyo et al. (2020) and Norimoto et al. (2020) find that the claustrum generates sharp ripples during slow-wave sleep. The application of carbachol (an agonist of both types of AChR) or 5-HT inhibit generation of sharp waves by the claustrum in brain slices (Norimoto et al., 2020). Because ACh levels are low during slow-wave sleep and high during wake states, cholinergic input to the claustrum may play a restrictive role, preventing the generation of sharp ripples during wake states while allowing such activity during sleep (Gais and Born, 2004; Nghiem et al., 2020). Although Norimoto et al. (2020) used an exogenous AChR agonist, carbachol, endogenous cholinergic input may also abolish sharp wave ripple generation in the claustrum *via* co-release of ACh and GABA inhibiting cortex-projecting claustrum neurons (Nair et al., 2021). This underscores the importance of studying neuromodulation caused by endogenous sources: precisely timed and highly local ACh release has specific effects that map onto only a subset of the phenomena observed with widespread application of pharmacological agents (Urban-Ciecko et al., 2018).

In summary, the opposing cholinergic gain mechanisms resulting from co-release of ACh and GABA may act as a canonical motif, allowing the claustrum to inhibit the cortex during attention and to generate sharp waves during sleep. In both cases, cholinergic input would play a permissive role but permitting different forms of brain activity.

### Research Gaps

Although we now have a potential mechanism and a hypothesis for cholinergic modulation in the claustrum, the behavioral role played by this permissive mechanism needs to be experimentally tested *in vivo*. In addition to actions within the claustrum, the cholinergic system can also cause attention-like effects by acting directly on the cortex. Thus, cholinergic actions on cortex and claustrum during attention will need to be disambiguated.

Besides opposing cholinergic modulation of claustrum projection neuron types, VIP interneurons also receive strong excitatory cholinergic input. In the claustrum, VIP interneurons are known to disinhibit projection neurons *via* their inhibitory action on other interneurons such as PV and SST interneurons (Graf and Augustine, 2019). However, the exact consequences of cholinergic modulation of VIP interneurons for claustrum network function remains unknown; predicting and testing its role will require detailed knowledge of claustral interneuron-projection neuron connectivity.

Endogenous cholinergic input in the claustrum appears to be mediated entirely by nAChRs. While Nair et al. (2021) did not observe mAChR activation, recent studies in the cortex indicate that endogenous acetylcholine activates mAChRs on dendrites rather than at the soma (Williams and Fletcher, 2019). The potential impact of cholinergic input on dendritic integration in the claustrum remains unknown and awaits further scrutiny.

## DA Signaling and The Claustrum

Dopaminergic modulation of the brain has received much attention, particularly in relation to the well-established role of DA in brain reward (Montague et al., 1996; Robinson et al., 2014). However, the role of DA in modulating the claustrum has received much less attention. Claustrum activity has been linked to rewarding, goal-directed behaviors such as the 5-choice serial reaction time task (5-CSRTT) and pup retrieval behaviors (Atlan et al., 2018; White et al., 2018, 2020), hinting at a possible role for DA signaling in the claustrum. To better understand dopaminergic regulation of the claustrum, we begin by describing the dopaminergic input that the claustrum receives and the types of DA receptors that are found within the claustrum. We will then consider the possible functions of DA regulation of the claustrum, particularly in the contexts of reward and brain disorders.

### Evidence of Dopaminergic Input and Receptors

Several lines of evidence point toward dopaminergic input to the claustrum in a variety of organisms. First, anterograde and retrograde tracers have been used to identify projections to the claustrum from the ventral tegmental area (VTA) and the substantia nigra pars compacta (SNpc), two of the main sources of dopaminergic modulation. Such evidence has been obtained in rodents (Lindvall et al., 1978; Zhang et al., 2001; Aransay et al., 2015; Beier et al., 2015; Zingg et al., 2018) as well as in reptiles (Norimoto et al., 2020). Conventionally, the VTA is part of the mesocorticolimbic system that is involved in aspects of higher cognition, while the SNpc afferents target subcortical regions and are involved in motor-related functions and learning (Grace et al., 2007; Luo and Huang, 2016; Klein et al., 2019). Although outputs of the VTA and SNpc are not exclusively dopaminergic (Beier et al., 2015; Morello and Partanen, 2015; Bouarab et al., 2019; Nagaeva et al., 2020), additional support for DA input to the claustrum comes from other studies. Tyrosine hydroxylase, an enzyme involved in DA synthesis, has been detected in innervation of the claustrum in humans (Sutoo et al., 1994), monotremes (Ashwell et al., 2004), rodents (Khlghatyan et al., 2019; Borroto-Escuela and Fuxe, 2020) and pigs (Pirone et al., 2018). However, because this enzyme is also found in neurons that release other types of catecholamines, such as epinephrine and norepinephrine (Purves et al., 2018), it is encouraging that terminals expressing DA transporter—which is specific to dopaminergic neurons—is found in the claustrum of humans (Ciliax et al., 1999), non-human primates (Al-Tikriti et al., 1995) and rodents (Ciliax et al., 1995; Freed et al., 1995; Delis et al., 2004).

An additional indication of DA signaling within the claustrum comes from studies of DA receptors. All five types of DA receptors, D1R to D5R, are metabotropic receptors and can be grouped into two classes. These two different classes of DA receptors produce different types of downstream responses. The first class, D1R-like, consists of D1R and D5R that predominantly activate Gs/olf proteins and increase intracellular cAMP levels. The second class, D2R-like, consists of D2R, D3R, and D4R that predominantly activate Gi/o proteins and decrease cAMP levels. D2R can undergo mRNA editing to yield to two variants: short and long forms. The molecular biology of DA receptors is a rich research area that is nicely summarized in multiple review articles (Beaulieu and Gainetdinov, 2011; Beaulieu et al., 2015; Mishra et al., 2018; Klein et al., 2019).

Many studies have used *in situ* hybridization and radioligand assays to localize D1R and D2R and to quantify levels of these receptors within the claustrum. Most of these studies agree that the claustrum has a high to moderate amount of D1R, albeit at levels much lower than found in the neighboring striatum. Such analyses have been done in rodents (Dawson et al., 1986; Savasta et al., 1986; Fuxe et al., 1987; Wamsley et al., 1989; Camps et al., 1990; Yoo et al., 2010; Borroto-Escuela and Fuxe, 2020; Hasbi et al., 2020; Terem et al., 2020), as well as in cats and monkeys (Camps et al., 1990). In contrast, studies of D2R receptor expression in the claustrum have yielded inconsistent results. Some report high D2R expression (Meador-Woodruff et al., 1991; Hall et al., 1996), while others indicate moderate to very low expression of D2R (Wamsley et al., 1989; Weiner and Brann, 1989; Camps et al., 1990; Mijnster et al., 1999; Khlghatyan et al., 2019; Borroto-Escuela and Fuxe, 2020; Hasbi et al., 2020; Terem et al., 2020). These diverse results could arise from the use of different species in different analyses, with D2R being more readily detected in primates (Camps et al., 1990; Meador-Woodruff et al., 1991; Hall et al., 1996). In addition, these results could be different due to isoform expression of D2R and type of radio-ligand tagged DA agonist/antagonist utilized (Camps et al., 1990). Nonetheless, dopaminergic modulation of the claustrum is likely to rely more on D1R than D2R as suggested by many studies in rodents (Fuxe et al., 1987; Wamsley et al., 1989; Weiner and Brann, 1989; Camps et al., 1990; Mijnster et al., 1999; Terem et al., 2020). The expression of other DA receptors within the claustrum has been examined less thoroughly: D3R and D4R have been detected in the claustrum, whereas D5R is likely to be absent (Meador-Woodruff et al., 1992; Suzuki et al., 1998; Mijnster et al., 1999; Borroto-Escuela and Fuxe, 2020).

### Possible Roles of Dopaminergic Modulation of the Claustrum

At present, only a few studies have examined the physiological actions of DA in the claustrum. Overall, DA seems to exert an inhibitory influence on the claustrum: Salerno et al. (1981) found that DA application inhibited a majority of claustral neurons in cats, while very few neurons were excited by DA. Unfortunately, neither the identity of the DA responsive neurons nor the DA receptors involved were identified. Considering the strong bias toward D1R expression in claustral neurons (Fuxe et al., 1987; Wamsley et al., 1989; Weiner and Brann, 1989; Camps et al., 1990; Mijnster et al., 1999), and the propensity of this receptor to increase neuronal excitability in other brain areas (Tritsch and Sabatini, 2012), the proportion of claustral neurons excited by DA is surprisingly low. More research is required to determine the exact mechanism of this claustral inhibitory DA response, be it through D1R/D2R homodimers or heterodimers (Hasbi et al., 2020), Gi/o-associated D2R-like receptors, or activation of inhibitory interneurons.

A recent study by Terem et al. (2020) has examined the role of D1R-expressing neurons in the claustrum in incentive salience. Incentive salience, otherwise known as “wanting”, is involved in reward learning as well as drug addiction (Robinson et al., 2014). Terem et al. (2020) demonstrated that D1R-expressing claustrum neurons are activated by cocaine administration, a condition known to involve DA signaling. Incentive salience was measured with a cocaine conditioned place preference (CPP) task: when the claustrum was inhibited during conditioning, cocaine CPP formation was disrupted. In a complementary experiment, CPP was produced when the claustrum was activated in a particular context. This action appeared to involve claustrum neurons projecting to frontal cortices, an area known to be involved in incentive salience (Robinson et al., 2014). Thus, the activity of these DA receptor-expressing projection neurons appears to be both necessary and sufficient for incentive salience.

Because the actions of cocaine could also involve targets beyond DA signaling (Carta et al., 2003; Filip et al., 2004), it is important to note that the study by Terem et al. (2020) did not directly examine the effects of DA on claustrum neurons. Nevertheless, this study is the first to implicate D1R-expressing claustrum neurons in incentive salience and complements other work that has indicated a role for the claustrum in rewarding, goal-directed behaviors (Atlan et al., 2018; Graf et al., 2020b; White et al., 2020). This strengthens the potential relationship between claustral dopaminergic modulation—perhaps coming from the VTA—and reward acquisition (Smythies et al., 2012; Zingg et al., 2018). It also provides additional support for a general role for the claustrum in salience (Graf et al., 2020b).

### Clinical Relevance of DA Modulation of the Claustrum

There are hints that DA modulation of the claustrum may also have clinical importance. In patients with Parkinson’s disease, the claustrum has significantly lower DA levels, presumably due to degeneration of SNpc (Sitte et al., 2017). How the effects of Parkinson’s disease on the nigroclaustral pathway differ from those of the nigrostriatal pathway remains unclear: Sitte et al. (2017) hypothesized that the nigroclaustral pathway relays sensorimotor information to the cortex faster than the nigrostriatal pathway and thereby could be responsible for the non-motor symptoms of Parkinson’s disease. Other studies have suggested that delusions associated with conditions such as bipolar disorder, dementia and depression occur due to hyperactivity of the mesolimbic pathway and the resultant aberration in salience, perhaps *via* recruitment of D2R in claustral neurons (Sitte et al., 2017). Whether delusions are caused by D2R activation in the claustrum remains unknown. Nevertheless, a role for claustrum DA signaling in delusions is plausible and consistent with the findings of Terem et al. (2020). More research, using analyses in both human and animal subjects, will be needed to clarify the clinical impact of DA modulation of the claustrum.

### Research Gaps

The possible modulation of the claustrum by DA is beginning to come into focus. However, much more research will be required to understand how DA affects claustral microcircuitry. As a start, it will be valuable to define whether DA acts on claustrum neurons by binding to D1R, D2R or D1R/D2R heterodimers. The physiological consequences of activating these receptors also needs to be determined. It will also be important to differentiate the effects of tonic versus phasic DA release in the claustrum: tonic DA release, which generates a relatively low and sustained elevation of DA concentration, preferentially affects D2-like receptors, while phasic DA release, which produces a higher and more transient rise in DA levels, preferentially affects D1-like receptors (Goto et al., 2007; Grace et al., 2007). Finally, possible differences in claustrum responses to DA released by the VTA versus the SNpc, during different tasks, must be examined to distinguish possible roles in reward versus motor function.

## Serotonergic Regulation of The Claustrum

From an evolutionary perspective, 5-HT is one of the oldest neuromodulators (Peroutka, 1995). In the mammalian brain, 5-HT is involved in a diverse range of processes, including sleep (Jouvet, 1999; Monti, 2010), learning and memory (Meneses, 2015; Zhang and Stackman, 2015), emotions (Altieri et al., 2013; Aznar and Klein, 2013; Bauer, 2015) and psychedelic drug action (Nichols, 2016; Canal, 2018). Although many of these processes overlap with the functions proposed for the claustrum, until recently serotonergic modulation of the claustrum was largely ignored. However, serotonergic modulation of the claustrum now is becoming an exciting topic. In this section, we summarize what is known about serotonergic modulation of the claustrum and the possible roles of such modulation in a variety of brain states.

### Evidence of Serotonergic Input and Receptors

Two midbrain nuclei are responsible for 5-HT release within the brain: the dorsal and median raphe nuclei (DRN and MRN respectively; Figure 1). Both have divergent and convergent targets throughout the brain (Hornung, 2003; Fernandez et al., 2016; Okaty et al., 2019). Anterograde and retrograde tracing experiments have established that both of these serotonergic nuclei project to the claustrum in cats (Rahman and Baizer, 2007) and rodents (Vertes, 1991; Peyron et al., 1998; Zhang et al., 2001; Zingg et al., 2018; Narikiyo et al., 2020). Most claustral serotonergic fibers originate in the dorsal and rostral DRN (Peyron et al., 1998; Muzerelle et al., 2016; Zingg et al., 2018) and are distinct from those projecting to the cortex (Rahman and Baizer, 2007). The synaptic terminals of DRN input to the claustrum are smaller and more spindle-shaped compared to terminals coming from MRN input (Wojcik et al., 2006). Both claustral projection neurons and interneurons receive serotonergic innervation (Baizer, 2001; Wojcik et al., 2006). As there are few claustral projections to the DRN and MRN (Peyron et al., 1998; Zhang et al., 2001; Ogawa et al., 2014; Pollak Dorocic et al., 2014), it is unlikely that the claustrum has a major reciprocal influence on the serotonergic system.

The mammalian 5-HT receptor (5-HTR) family consists of seven subfamilies. 5-HTRs that activate Gi/o proteins include the 5-HTR-1 subfamily—subtypes 5-HTR-1A to 1F—as well as 5-HTR-5 subfamily members 5-HTR-5A and 5-HTR-5B. The 5-HTR-2 subfamily includes three receptors—5-HTR-2A, 5-HTR-2B, and 5-HTR-2C—that activate Gq proteins. The remaining metabotropic 5-HTRs, which are subtypes 5-HTR-4S, 5-HTR-4L, 5-HTR-6, and 5-HTR-7, activate Gs proteins. 5-HTR-3 subfamily members are unique because they are ionotropic 5-HTRs that are structurally similar to nAChRs (Thompson et al., 2010). Unlike metabotropic 5-HTRs, 5-HTR3s are expressed exclusively in a subset of brain interneurons; indeed, expression of 5-HTR3s defines this interneuron type (Lee et al., 2010; Rudy et al., 2011; Koyama et al., 2017). Comprehensive descriptions of 5-HTRs can be found in numerous reviews (Ciranna, 2006; Nichols and Nichols, 2008).

Altar et al. (1985) apparently were the first to identify 5-HTRs in the claustrum. They observed an enrichment of 5-HT binding to membrane fractions from the rat claustrum, establishing the presence of membrane-associated 5-HTR in the claustrum. Subsequent work identified the 5-HTR types that are present in the claustrum. These studies employed 5-HTR agonist and antagonist radioligands (e.g., ketanserin); one caveat of such analyses is the known off-target effects of these agents (Aloyo and Harvey, 2000; Canal, 2018). The consistent conclusion of such studies is that 5-HTR-2A and 5-HTR-2C are highly expressed in the claustrum of several species (Dawson et al., 1986; Mengod et al., 1990; Pompeiano et al., 1994; Wright et al., 1995; Ward and Dorsa, 1996; Hamada et al., 1998; Rioux et al., 1999; Kinsey et al., 2001; Olaghere da Silva et al., 2010; Gawliński et al., 2019). Remarkably, the claustrum has been found to have the highest density of 5-HTR-2A in the entire mouse brain (Rioux et al., 1999). Other studies have found either low or high expression of 5-HTR-1A in the claustrum; the labeling efficiency of 5-HTR-1A antagonists is reportedly better than that of 5-HTR-1A agonists (Mengod et al., 1990) and rats might have lower levels of 5-HTR-1A (Wright et al., 1995) in comparison to monkeys (Pazos et al., 1987), humans (Mengod et al., 1990) and tree shrews (Palchaudhuri and Flügge, 2005). While 5-HTR-1F are also abundant in the guinea pig claustrum (Mengod et al., 1990; Bruinvels et al., 1994), other metabotropic 5-HTRs—such as 5-HTR-1D, 5-HTR-2B, 5-HTR-4, 5-HTR-5A, 5-HTR-5B, 5-HTR-6, and 5-HTR-7—are expressed at low levels in the claustra of a variety of species, including rodents (Mengod et al., 1990; Bruinvels et al., 1994; Wright et al., 1995; Ward and Dorsa, 1996; Bonaventure et al., 1997; Gérard et al., 1997; Kinsey et al., 2001), non-human primates (Mengod et al., 1990; Bruinvels et al., 1994), rabbits, and humans (Mengod et al., 1990). 5-HTR-1B levels are low in rat and moderate in guinea pig brains (Bruinvels et al., 1994; Bonaventure et al., 1997). Some studies report a moderate to low expression of 5-HTR-3 in the claustrum (Gehlert et al., 1991; Carrillo et al., 2010). Sparse expression of 5-HTR-3 is expected because these receptors are found only in a subpopulation of claustrum interneurons (Lee et al., 2010; Rudy et al., 2011; Koyama et al., 2017), including VIP interneurons that are approximately 4% of all claustrum neurons (Graf et al., 2020a). No study has systematically characterized 5-HTR expression in claustral interneurons.

### Actions of 5-HT and Its Agonists in the Claustrum

At the microcircuit level, whole-cell patch clamp recordings in claustrum slices have established that 5-HT overall exerts an inhibitory effect on the claustrum (Wong and Augustine, 2019). 5-HT produces a prolonged inhibition of claustrum projection neurons that lasts for seconds. Conversely, interneurons exhibit diverse responses to 5-HT: some are excited while others are inhibited by an action of 5-HT. Together, the net effect of 5-HT responses from claustral projection neurons and interneurons reduces claustral output.

Psychedelic drugs—such as LSD and psilocybin—exert their actions by binding to 5-HTR-2 and other targets (Wacker et al., 2017). Because the claustrum highly expresses 5-HTR-2A and sends dense projections throughout the brain, it is ideally positioned as a potential target for psychedelic action and consequent cortical network destabilization (Martin and Nichols, 2016; Nichols, 2016). For these reasons, activation of the claustrum by 5-HTR-2A has been hypothesized to contribute to the actions of psychedelic drugs. Martin and Nichols (2016) have shown that the psychedelic compound DOI, which is an agonist of 5-HTR-2A and 5-HTR-2C, activates claustrum neurons. Specifically, they found that c-Fos levels, a surrogate of neuronal electrical activity, are sharply increased in claustrum neurons in response to DOI. They also found an associated internalization of 5-HTR-2A, which is consistent with an action of DOI on these receptors. The effects of psilocybin, a 5-HTR-2A agonist, in the human claustrum were recently studied by Barrett et al. (2020). Psilocybin decreased the activity of the claustrum, but not the activity of neighboring structures such as insula or putamen. This effect was correlated both with self-reported measures of the perceived strength of psilocybin and with measures of mystical experiences, such as ineffability. Psilocybin was also found to alter claustral functional connectivity within various cognition-related networks. In particular, the connectivity of the fronto-parietal task network with both left and right claustra were altered; psilocybin also attenuated the connectivity of the right claustrum with the default mode network. Collectively, these findings indicate that much more work is needed to clarify the action of psychedelics on the claustrum and to reconcile measurements made on molecular, microscopic and macroscopic levels.

Previous work has suggested that the claustrum is involved in sleep (Hong et al., 2009; Renouard et al., 2015; Jansen et al., 2019; Narikiyo et al., 2020). A recent landmark study by Norimoto et al. (2020) has advanced this suggestion by providing key evidence that claustrum 5-HT signaling is important for sleep. They found that the reptilian claustrum is involved in the generation of slow waves during sleep. This effect is regulated by 5-HT, because uncaging of 5-HT in claustrum slices during sleep-like states abolished slow-wave activity. Brain 5-HT levels are lower during sleep than during wakefulness (Jouvet, 1999; Portas et al., 2000; Monti, 2010); thus, sleep may be enabled by the resulting claustrum slow-wave activity. The inhibition of claustrum slow-wave activity by 5-HT during the awake state is primarily mediated by 5-HTR-1D, with lesser contributions by 5-HTR-1A and 5-HTR-2C. This corroborates claustral inhibition by 5-HT observed by Wong and Augustine (2019).

### Consciousness and Serotonergic Modulation of the Claustrum

Crick and Koch (2005) proposed that the claustrum serves as the “seat of consciousness”. Several lines of evidence supporting the claustrum-consciousness connection have been derived from electrical stimulation studies. For instance, electrical stimulation of the claustrum induces sleep bouts and/or unresponsiveness in animals (Gabor and Peele, 1964; Vakolyuk et al., 1983) and increases anesthetic depth (Pavel et al., 2019). Additionally, a loss of consciousness (LOC) was reported in one human subject in response to electrical stimulation near the claustrum (Koubeissi et al., 2014), though a follow-up study was unable to replicate this intriguing finding in five subjects (Bickel and Parvizi, 2019). Thus, we do not know what influences the claustral “consciousness conductor” in a normal brain.

Perhaps 5-HT actions within the claustrum play a role in maintaining consciousness. To identify loci associated with LOC, Snider et al. (2020) used lesion network mapping in humans to uncover an anticorrelation of activity between the DRN and claustrum that is strongly linked to LOC. Such an anticorrelation could be explained by evidence from the work of Wong and Augustine (2019) and Norimoto et al. (2020) clearly demonstrating that 5-HT inhibits the claustrum. Because various phases of sleep are considered to represent different states of consciousness (Laureys, 2005; Kraehenmann, 2017), it is possible that serotonergic inhibition of the claustrum is also involved in regulating consciousness. These studies suggest that LOC is caused by disinhibition of the claustrum resulting from loss of serotonergic inhibition from the DRN, and harkens back to the seminal proposal by Crick and Koch (2005). More research clearly is needed to determine whether the claustrum has an actual role in consciousness, as advocated by Crick and Koch (2005), and whether 5-HT participates in such a role. Further, consciousness is a complex phenomenon and there undoubtedly are many other contributors beyond serotonergic inhibition of the claustrum (Zhao et al., 2019; Snider et al., 2020).

### Link Between Neuropsychiatric Disorders and the Claustrum

Many neuropsychiatric disorders are associated with 5-HT imbalances in the brain (Marek et al., 2003; Nordquist and Oreland, 2010; Lin et al., 2014). One of the main approaches to manage such disorders is to administer 5-HT reuptake inhibitors, drugs that target 5-HTR, and—more recently—psychedelics (Marek et al., 2003; Rucker et al., 2018). Similarly, the claustrum has also been implicated in many neuropsychiatric disorders: claustral volume is smaller in humans with bipolar disorder (Selvaraj et al., 2012), depression and schizophrenia (Bernstein et al., 2016). The human claustrum also expresses depression-related genes (Ibrahim et al., 2019) and its activity is altered across multiple neuropsychiatric disorders (Farruggia et al., 2020). Moreover, the claustrum has been implicated in anxiety (Smith et al., 2019b; Niu et al., 2020). Given that the claustrum has a high density of 5-HTRs and is influenced by serotonergic psychedelics, the claustrum can be considered as an emerging target for these neuropsychiatric disorders.

### Research Gaps

5-HT is important for many complex and essential brain functions. When attempting to map the known roles of 5-HT onto the many proposed functions of the claustrum, it is hard to pinpoint a precise function for serotonergic modulation of the claustrum. The newly established link between the claustrum, sleep and 5-HT in reptiles (Norimoto et al., 2020) demands additional research to determine the applicability of these findings to the mammalian brain. Furthermore, serotonergic regulation of the claustrum during wake states should be explored to understand whether such modulation plays a role in other 5-HT-associated functions, for example, memory and emotion. Finally, the role of the claustrum in psychedelic drug action should be examined more systematically to provide insights into psychedelic-induced brain states and possibly pave the way for psychedelic-assisted therapies for neuropsychiatric disorders such as depression.

## Other Neuromodulators

The information above makes clear that the claustrum, like many other brain regions, is likely to be influenced by numerous neuromodulators during various brain states. Although we have focussed on ACh, DA and 5-HT, the claustrum may also be regulated by other neuromodulators, which could share converging effector pathways (Nadim and Bucher, 2014). A holistic view of this diversity of neuromodulators is needed to develop a comprehensive understanding of claustral function in various brain states. Early hints about the roles of other neuromodulators come from studies of receptor localization and analyses of claustral innervation by axonal projections containing these neuromodulators.

### Norepinepherine

The potential for norepinephrine (NE) to modulate the claustrum has garnered some attention. NE-positive fibers are found in the claustrum (Baizer, 2014; Pirone et al., 2018) and the expression and possible function of NE receptors in the claustrum have been theme of several studies (Palchaudhuri and Flügge, 2005; Baizer, 2014; Pirone et al., 2018; Smith et al., 2019a; Borroto-Escuela and Fuxe, 2020). Lower amounts of NE are found in the claustrum of Parkinson’s disease patients, suggesting a potential role in the etiology of this motor disorder (Sitte et al., 2017). In summary, although there are suggestions of a role for NE in modulation of the claustrum, currently there are no compelling ideas about what this role might be. Clearly there is a need for physiological analyses of the actions of NE on the claustrum.

### Neuropeptides

Among the genes highly expressed in claustral neurons, Wang et al. (2017) identified SST receptor 2 (SSTR2) and opioid receptor kappa 1 (KOR1). These two receptors can also heterodimerize, potentially increasing their signaling capabilities (Borroto-Escuela and Fuxe, 2020). KOR1 is known to be involved in chronic cocaine exposure (Collins et al., 2002) and could complement the role of claustral D1R in acute cocaine responses (Terem et al., 2020). The dense expression of KOR1 in the claustrum (Chen et al., 2020) could be involved in delusions caused by the KOR1 agonist salvinorin-A (Patru and Reser, 2015). KOR1 also has been proposed to play a role in orchestrating consciousness (Stiefel et al., 2014).

### Neuromodulator Co-release

Neuromodulators can also be co-released along with conventional neurotransmitters. In the claustrum, it has recently been found that the inhibitory transmitter GABA is co-released along with ACh from forebrain cholinergic system axons (Nair et al., 2021). Co-release of 5-HT and glutamate may also occur: retrograde tracing experiments by Zingg et al. (2018) and the Allen Institute (Experiment 478995566) identify DRN and MRN neurons that may represent serotonergic neurons that also contain the glutamate vesicular transporter, vGluT3, involved in glutamate release (Okaty et al., 2015, 2019; Huang et al., 2019; Ren et al., 2019). Claustral interneurons containing neuropeptide, such as SST and VIP (Graf et al., 2020a; Marriott et al., 2020), could also co-release these peptides along with GABA. Claustral co-release of any neuropeptide has yet to be demonstrated; neuropeptide release typically requires high-frequency activity, so an analysis of neuropeptide release will require such stimulation paradigms (Liguz-Lecznar et al., 2016; Mazuski et al., 2018). The possible physiological roles of co-released neuromodulators certainly merit further attention.

## A Unified Picture of Neuromodulator Control of The Claustrum

Given this rich landscape of neuromodulation, how does the claustrum contribute to brain function by integrating these diverse, dynamic, and sometimes antagonistic signals? Pondering this question is all the more challenging because the fundamental roles of the claustrum are still open to debate. Nonetheless, in this section we will tackle the question.

**Figure 2:**
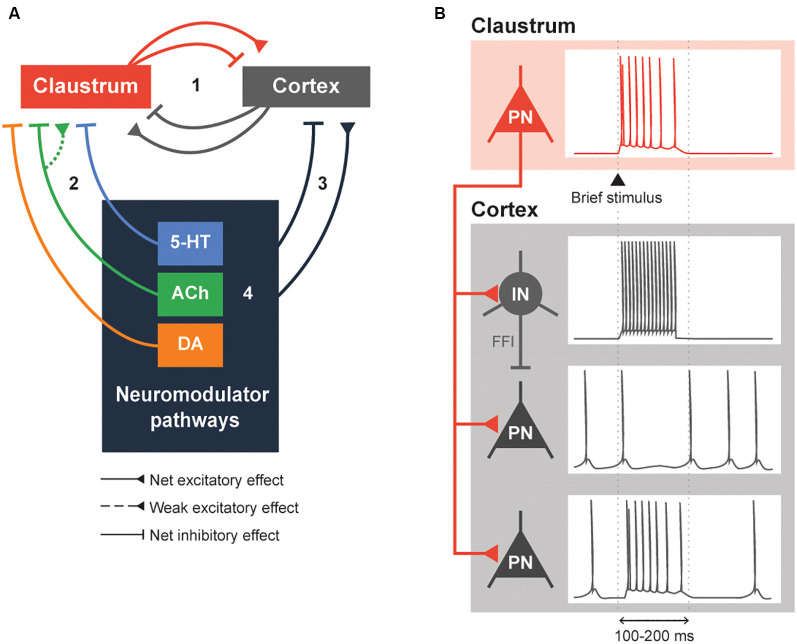
Neuromodulation of the claustrocortical loop. **(A)** A simplified model consisting of the claustrum, the cortex and various neuromodulatory structures that release serotonin (5-HT), acetylcholine (ACh) and dopamine (DA). The claustrocortical loop (Pathway 1) consists of the claustrum and the cortex causing either a net excitation or inhibition of each other. Both the claustrum (Pathway 2) and cortex (Pathway 3) receive heavy input from the neuromodulator pathways. These neuromodulator pathways are also capable of regulating themselves (black box 4). **(B)** An illustration of claustrum input to the cortex. A claustral projection neuron (PN) receiving a brief stimulus will fire action potentials, which sends excitatory input into cortex. The cortical interneuron (IN) fires action potentials due to the excitation and subsequently inhibits the cortical PN. The cortical PN, receiving feed-forward inhibition through the cortical IN and excitation by the claustral PN, might initially fire action potentials and will remain silent during the period of inhibition by the cortical IN. Conversely, a subset of cortical PN will be directly excited by the claustral PN. These changes in cortical PN and IN activity would typically last for 100–200 ms or until claustral PN activity is diminished.

Our answer begins with the simplified network, consisting of four pathways, shown in Figure 2A. Within a claustrocortical loop (Pathway 1), the claustrum may exert net inhibitory effects through feed-forward inhibition (FFI) that is mediated by claustrum excitation of local cortical interneurons (Ptito and Lassonde, 1981; Tsumoto and Suda, 1982; Salerno et al., 1984; Jackson et al., 2018; Narikiyo et al., 2020) and/or possibly net excitation, as suggested by more recent evidence (Fodoulian et al., 2020). This loop also includes excitatory monosynaptic input that claustral projection neurons and interneurons receive from various cortical regions (Kim et al., 2016; White and Mathur, 2018b; White et al., 2018; Chia et al., 2020); whether this combined input exerts a net inhibitory or excitatory effect on the claustrum remains unknown. Pathways 2 and 3 indicate potential modulation of the claustrocortical loop by neuromodulators. The cholinergic and serotonergic systems exert a net inhibitory influence on the claustrum, albeit at differing timescales. The ACh system, *via* the co-release of GABA, exerts fast inhibitory effects mediated by ionotropic GABA receptors. This may enable the claustrum to control shorter-lasting brain states such as attention, while 5-HT responses are largely mediated by metabotropic 5-HTR and thus could mediate longer-lasting brain states, such as sleep. Although understudied, DA regulation could inhibit the claustrum rapidly or slowly, depending on the DA receptors involved. Finally, neuromodulator pathways can influence each other (Pathway 4): for example, 5-HT release from the DRN attenuates DA release from the VTA (Conio et al., 2020) and ACh is known to increase the release of a variety of neuromodulators, including DA and 5-HT (Picciotto et al., 2012).

Claustral projections to the cortex share some similarities with the neuromodulatory projections arising from the cholinergic basal forebrain, dopaminergic VTA and SNpc, and serotonergic raphe nuclei: in all cases, multiple cortical regions are targeted, which enables widespread control of the cortex (Torgerson et al., 2015; Wang et al., 2017, 2019; Marriott et al., 2020; Narikiyo et al., 2020). Then what is the logic of having both types of pathway project to the cortex? One distinction appears to be the time course of cortical regulation. Activation of the claustrum can cause a brief inhibition of cortical activity that lasts 100–200 ms (Figure 2B; Cortimiglia et al., 1982; Salerno et al., 1984; Jackson et al., 2018; Narikiyo et al., 2020). This arises from strong FFI of cortical projection neurons by local interneurons, which serves to attenuate the initial excitation provided by excitatory claustrum input (Jackson et al., 2018; Narikiyo et al., 2020). More recently, claustral neurons also were shown to cause a brief net excitation of cortical neurons (Fodoulian et al., 2020). Unlike the claustrum, neuromodulators can exert their effects on either a short timescale or a long timescale, depending on the postsynaptic receptors involved. Further, neuromodulatory systems heavily rely on volume transmission—diffusion of neuromodulators through the extracellular space—to modulate entire populations of spatially contiguous neurons in the central nervous system (Fuxe et al., 2010). These differences distinguish claustral and neuromodulatory input.

Another difference between the claustrum and neuromodulatory system is the degree of feedback from the cortex. The paucity of afferents makes it unlikely that the claustrum and cortex exert much direct feedback control on neuromodulatory brain areas (Peyron et al., 1998; Zhang et al., 2001; Polack et al., 2013; Ogawa et al., 2014; Beier et al., 2015). In contrast, it is well-established that the claustrum is bilaterally and bidirectionally connected to most cortical regions (Torgerson et al., 2015; Wang et al., 2017, 2019); any changes in cortical output should swiftly influence claustral activity. Therefore, as compared to neuromodulatory pathways, the dynamics of the claustrocortical loop should allow for temporally precise recruitment of neuronal ensembles across the cortex.

By virtue of the broad distribution of neuromodulatory axons throughout the brain, neuromodulators will simultaneously affect many brain regions. How does neuromodulation affect the claustrocortical loop? As illustrated in Figure 2A, we can anticipate complex interactions between neuromodulatory structures, the cortex and the claustrum. The precise effect of neuromodulation is likely dependent on the connectivity between the different cortical areas and the claustrum. For instance, neuromodulation of the claustrum-anterior cingulate cortex (ACC) loop should be very different from that of the claustrum-retrosplenial cortex (RSC) connection: unlike the ACC, the RSC receives dense monosynaptic excitatory input from the ipsilateral claustrum but does not send afferents to the claustrum (Wang et al., 2017; Brennan et al., 2020; Marriott et al., 2020). Additionally, there may be differences in the types of postsynaptic neurons that are targeted by neuromodulator pathways, which could differentially affect responses in the claustrum and elsewhere (Beier et al., 2015; Huang et al., 2019; Nair et al., 2021). At present, most research has focused on a claustrum-centric model, where neuromodulators primarily act upon the claustrum, which then inhibits the cortex (Martin and Nichols, 2016; Graf et al., 2020b; Norimoto et al., 2020; Terem et al., 2020; Nair et al., 2021). More research is required to understand whether neuromodulatory input to the cortex, including presynaptic modulation (Nadim and Bucher, 2014), affects claustral function similarly.

Brain states, such as those occurring during sleep and psychedelic-altered conditions, are characterized by synchronized brain activity. These states apparently arise from the coordinated action of long-lasting neuromodulators (Kringelbach and Deco, 2020; McCormick et al., 2020). To better understand the mechanisms producing brain states, neuronal activity must be correlated with observable behaviors. While still at an early stage, available studies of the claustrum have demonstrated patterns of claustral activity associated with different brain states, including those of wake and sleep states. A majority of claustral neurons fire at a low rate during wake states (Jankowski and O’Mara, 2015; Narikiyo et al., 2020; Reus-García et al., 2020). During tasks that requires attention and/or cognition, such as the 5-CSRTT and attentional set-shifting task, claustral activity exhibits a gradual increase over a second before a reward and scales with the cognitive load of the task (White et al., 2018, 2020; Fodoulian et al., 2020). In comparison to wake states, claustrum activity increases during sleep (Narikiyo et al., 2020; Norimoto et al., 2020). State-related differences could also influence the effect of claustral output; cortical slow-wave ripples caused by claustral stimulation appear smaller in magnitude during wake states than in sleep states (Narikiyo et al., 2020). These differences in claustral activity and output during distinct brain states may arise from differences in neuromodulator actions in the claustrum.

At a more macroscopic level, neuromodulation can select for the activity of specific brain networks during different brain states. This can be reflected in changes in intrinsic connectivity networks (ICNs): claustral functional connectivity to various brain regions can be altered in different brain states. This has been established by many studies demonstrating that pharmacological interventions, anatomical lesions and sleep perturb both claustrum activity and claustrum connectivity to various ICN nodes (Hong et al., 2009; Chau et al., 2015; Barrett et al., 2020; Snider et al., 2020). However, the precise change in claustral functional connectivity relative to various ICN nodes in different brain states and neuromodulators remains an open question.

**Figure 3:**
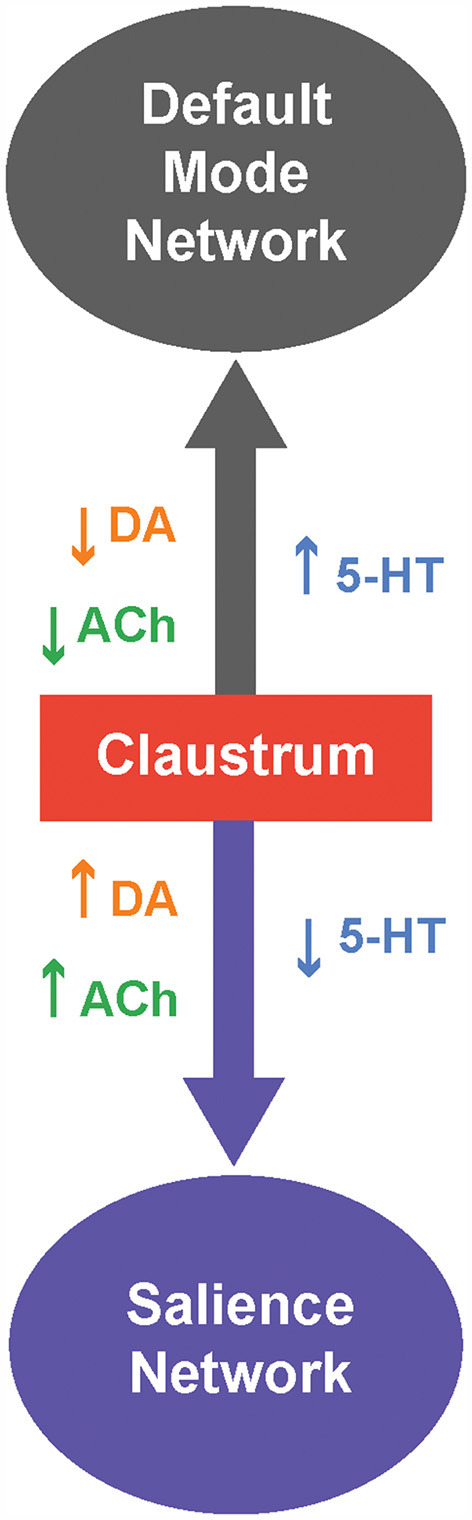
Neuromodulatory control of the claustrum and intrinsic connectivity networks (ICNs). The claustrum serves as a switch between two ICNs: The default mode network (DMN) and salience network (SAN). When 5-HT levels are high,and levels of DA and/or ACh are low, the claustrum is likely inhibited and this favors activation of the DMN. Conversely, when 5-HT levels are low, and levels of DA and/or ACh are high,the claustrum permits activation of the SAN.

We can take cues from the ICNs the claustrum is involved in and from the responses of these ICNs to neuromodulators to form a unified picture of neuromodulator control of the claustrum. The claustrum is thought to participate primarily in two ICNs: the salience network (SAN) and the default mode network (DMN; Figure 3; Smith et al., 2019b). Specifically, the claustrum serves as a link between the anterior insula and the ACC (Wang et al., 2017; Chia et al., 2017, 2020; Qadir et al., 2018; Zingg et al., 2018; Krimmel et al., 2019; Rodríguez-Vidal et al., 2019; Smith et al., 2019a), two very prominent components of the SAN (Menon and Uddin, 2010). As the claustrum also is connected within the DMN, it is ideally positioned to process sensory-limbic information and use this information to toggle in and out of the DMN like a switch; the claustrum is linked to the recruitment of the SAN and disengagement of the DMN (Krimmel et al., 2019; Rodríguez-Vidal et al., 2019; Smith et al., 2019b). Such toggling is likely a result of selection of different subsets of claustral projection neurons with differing projection patterns (Wang et al., 2019; Chia et al., 2020; Graf et al., 2020a; Marriott et al., 2020) during a particular state. Thus, when the claustrum is activated in the SAN, other task-positive networks such as the fronto-parietal network can be activated (Menon and Uddin, 2010).

Which neuromodulators could permit the claustrum to switch from the DMN to the SAN? Several human and murine studies have investigated how neuromodulators, particularly 5-HT, DA and ACh, strengthen and weaken these ICNs. A serotonin-dopamine antagonism can be seen between the SAN and DMN; in humans, DA from the VTA strengthens the SAN while 5-HT strengthens the DMN (Conio et al., 2020). Such antagonism is also apparent in the observation that the serotonergic psychedelic psilocybin strengthens DMN networks and dampens DA-related networks in mice (Grandjean et al., 2021). A possible mechanism for 5-HT-associated strengthening of the DMN could be 5-HT inhibition of the claustrum (Wong and Augustine, 2019; Norimoto et al., 2020), which would attenuate claustral activity and connectivity with the DMN (Barrett et al., 2020), thereby strengthening the DMN (Krimmel et al., 2019; Rodríguez-Vidal et al., 2019; Smith et al., 2019b). In contrast to 5-HT, DA from the VTA strengthens the SAN. Perhaps VTA-related DA activation of the claustrum *via* D1 is required for salience (Terem et al., 2020). However, this hypothesis is inconsistent with the observations of Salerno et al. (1981) that most claustral neurons are inhibited by DA. ACh exerts effects on the DMN and SAN that are similar to those of DA. In humans, ACh activation of nAChRs weakens the DMN and strengthens ICNs, such as the SAN, that are involved in cognition (Kokaz et al., 2020). Similarly chemogenetic activation of the basal forebrain of rats decreases the DMN and increases task-positive ICNs (Peeters et al., 2020). This is consistent with the ability of cholinergic modulation to dynamically bias claustrum output towards cortical targets, relative to subcortical targets (Nair et al., 2021), and could enable attention through the SAN (Atlan et al., 2018; Jackson et al., 2018; Smith et al., 2019b; Fodoulian et al., 2020). To summarize, brain states associated with differing neuromodulatory influences can cause the claustrum to switch between the SAN and DMN. Moreover, considering both the microscopic and macroscopic levels of neuromodulation can help to resolve and spur questions on the functions of claustrum neuromodulation.

We are at the earliest stages of unravelling the influence of different neuromodulators on claustral function in various brain states. As the general function and release of most common neuromodulators are well-defined, brain state transitions may serve as an informative point of access to understand claustrum neuromodulation (Kringelbach and Deco, 2020). This could permit correlation of changes in claustral activity with the release of neuromodulators in the claustrum during transitions among brain states. Hence studies such as those of Norimoto et al. (2020), where sleep was used as a proxy for such a transition, may be particularly useful for determining how claustral activity is regulated by neuromodulators. This approach would also hint at how the claustrum could subserve higher functions that are linked to a particular brain state. Specifically, various brain states could help explain why the claustrum seems to be involved in multiple functions ranging from attention to sleep.

## Conclusions

We propose that rich neuromodulatory input confers upon the claustrum a unique ability to rapidly switch cortical activity dynamics across a wide range of structures during a variety of brain states. While neuromodulation is usually thought to enable diffuse regulation of brain structures, having the claustrum as a focus may enable more precise neuromodulatory control of cortical regions, especially those involved in higher cognition. Hence, the claustrum could genuinely serve as a cortical conductor, fine-tuning its inhibitory and/or excitatory tempo based on the current brain state.

With this proposal in mind, we will close by listing several questions that could guide future investigations of claustrum neuromodulation:

(1) Neuromodulation of the claustrum: given that there are at least eight different types of claustral neurons (Kim et al., 2016; Chia et al., 2017; White and Mathur, 2018a; Graf et al., 2020a), how does each respond to a given neuromodulator? If there are neuron-specific differences in neuromodulator responsiveness, as has already been observed for the cases of 5-HT and ACh, what is the net effect of each neuromodulator on claustrum microcircuitry and output?
(2) Inputs into the claustrum: what are the net effects of various cortical and subcortical inputs on the claustrum? How do these inputs change the output of the claustrum and how are their influences regulated by neuromodulators?
(3) Outputs of the claustrum: given the various cell types present in the claustrum, are there differences in the dynamics and output firing properties of these claustral subpopulations? Is cortical local circuitry differentially affected by these subpopulations?
(4) Neuromodulation of the claustrocortical loop: are there differences in the timing or amount of neuromodulator release between the cortex and the claustrum? Do claustral responses to neuromodulators, such as DA and 5-HT, differ when these modulators are released in a tonic versus phasic fashion? How do neuromodulators regulate the claustrum during different brain states, for example, sleep-wake states? Is the activity and functional connectivity of the claustrum altered by neuromodulators and/or by different brain states?

We look forward to seeing at least some of these questions answered in the near future.

## Author Contributions

All authors contributed to the article and approved the submitted version.

## Conflict of Interest

The authors declare that the research was conducted in the absence of any commercial or financial relationships that could be construed as a potential conflict of interest.
